# Avoidance of bile duct injury in laparoscopic cholecystectomy with feasible intraoperative resources: A cohort study

**DOI:** 10.3892/br.2024.1798

**Published:** 2024-06-05

**Authors:** Deari A. Ismaeil

**Affiliations:** 1 Department of Surgery, College of Medicine, University of Sulaimani, Sulaimani, Kurdistan 46001, Iraq; 2 Scientific Affairs Department, Smart Health Tower, Sulaimani, Kurdistan 46001, Iraq

**Keywords:** bile duct injury, conversion, critical view of safety, Rouviere's sulcus, laparoscopic cholecystectomy

## Abstract

Laparoscopic cholecystectomy (LC) is one of the most commonly performed surgeries and is considered the standard treatment for cholelithiasis. However, it is associated with a risk of bile duct or hepatic artery injuries. This study evaluated the safety of LCs and the conversion rate (CR) by achieving a critical view of safety (CVS) and identification of Rouviere's sulcus (RS). This was a single-group cohort study that included consecutive patients undergoing LC at Smart Health Tower (Sulaimani, Iraq) from January 2021 to January 2023. The data were prospectively collected from patients' profiles or surgical notes within the hospital's database. A total of 419 patients underwent LC, of which females were the predominant gender (78.5%). The mean and median ages of the cases were 46.3±15.8 and 45 years, with a range of 2-90 years, respectively. The most common indications for surgery were biliary colic (69.5%), followed by acute cholecystitis (23.9%). The duration of the operations was significantly shorter for cases in which the CVS (45.6±17.9 min) or identification of RS (45.6±18.6 min) was achieved compared to those where the CVS (63.7±27.7 min) or RS (50.7±21.7 min) was not observed. Surgeries for patients with both CVS achievement and RS identification were also significantly less time-consuming (44.3±17.6) than counterparts (53.3±22.6). Among the cases without CVS achievement or RS identification (n=97, 23%), eight (8.2%) had adhesions, 12 (12.4%) had a distended gallbladder (GB) and 10 (10.3%) had thick GB walls. In addition, four (4.1%) experienced GB perforation, two (2.1%) had bleeding and one (1%) had stone spillage. There was no conversion. The achievement of CVS and identification of RS are practical landmarks in performing safe LC and decreasing the CR.

## Introduction

Laparoscopic cholecystectomy (LC) is one of the most common and widely performed surgeries worldwide, serving as the gold standard treatment for cholelithiasis (1). The most concerning complications in LC are bile duct injuries (BDI) or hepatic artery injuries, hemorrhage and subhepatic abscess (2,3). The rates of BDI have increased since the introduction of LC (4). Several factors have been shown to increase susceptibility to biliary injury during LC, including obesity, male gender, advanced age, the presence of gallbladder (GB) adhesions, inflammation or infection, hemorrhage, anomalous anatomical configurations and the skill level of the surgeon (5). In general, BDI presents a significant complication of LC and is associated with notable perioperative morbidity and mortality, reduced long-term survival, diminished quality of life and an increased frequency of legal proceedings (6). Misinterpreting the configuration of biliary anatomy has been pinpointed as the fundamental trigger for injury in ~70-80% of instances (6). Training and experience have a crucial role in reducing BDI, yet even the most experienced surgeons can still make incorrect dissections and inadvertently damage the bile ducts (7). Enhancing the learning curve and gaining experience have been advocated to ensure the safety of LC. These efforts encompass the establishment of the critical view of safety (CVS) as a method for identifying the cystic duct and cystic artery during LC. This involves clearing the Calot's triangle (CT) of fat and fibrous tissue, isolating the lower portion of the GB from the cystic plate and ensuring that only two structures are visible as they enter the GB (8). Furthermore, the identification of Rouviere's sulcus (RS) during LC can help improve safety (9). While the utilization of intraoperative cholangiography (IOC) has been proposed to enhance the visualization of biliary anatomy and reduce bile duct injuries (BDIs), this approach is also accompanied by potential morbidity and mortality. It requires the availability of suitable facilities, necessary equipment and experienced practitioners (10).

The present study aimed to evaluate the safety of LCs and the conversion rate (CR) through achieving CVS and identification of RS. The study was written according to the Strengthening The Reporting Of Cohort Studies in Surgery 2019 guideline (11) and cited studies were checked to avoid citing suspicious information in predatory journals (12).

## Patients and methods

### Study design

This was a single-group cohort study that enrolled consecutive patients who underwent LC at Smart Health Tower (Sulaimani, Iraq) from January 2021 to January 2023. The study had been ethically approved by the Ethics Committee of the College of Medicine at the University of Sulaimani (Sulaimani, Iraq; approval no. 85). All participants provided consent to participate in this study and to the publication of any related data.

### Data collection

The data were collected prospectively from patients' medical records or surgical notes recorded by the surgeon within the hospital's database. The data included patient demographics, clinical presentations, prior admissions, operative findings, attainment of CVS and RS, operation duration, intraoperative events and postoperative complications that were documented throughout the one-month follow-up period. The follow-up was based on weekly visits of the patients to the hospital. The operation time was calculated from making the first incision to the closing of the last incision.

### Eligibility criteria

All cases that were candidates for LC from January 2021 to January 2023 at Smart Health Tower, regardless of surgery indication, were included in this study. Patients who were not deemed suitable for general anesthesia, and those who did not provide consent either to undergo the procedure or to participate in the study, were excluded.

### Intervention

A senior gastrointestinal surgeon conducted all of the procedures. The procedures were performed using the American method and the standard four-port technique (13). For patients with upper abdominal or subcostal scars, the infra-umbilical open technique was employed following insufflation of CO_2_ through a Veress needle or visual entry cannula trocars (VisiPort). In cases of long midline scarring, as well as multiple scars, a non-standard port-site approach was chosen for the initial port. This involved utilizing 5-mm epigastric or left subcostal (Palmer's point) sites with a 5-mm telescope. Adhesions between the GB and the omentum or bowel loops were released as necessary, utilizing blunt or sharp dissection techniques with the assistance of Ligasure (Medtronic Ltd.). The thick distended GB or empyema was decompressed before grasping. The standard procedure involved consistently achieving the CVS and identifying the RS whenever feasible. Adequate exposure of the hepato-cystic triangle's anatomy was ensured, and in cases of unclear anatomy or Mirizzi syndrome, a subtotal cholecystectomy was performed (only 2 cases).

### Primary and secondary outcomes

The primary outcome was to determine the maximum safe extent to which LC could be performed with minimal complications using perioperative strategies such as practicing CVS and identifying RS. The secondary outcome was to ascertain the extent to which CRs could be reduced by implementing these techniques.

### Statistical analysis

Data organization and coding were performed using Excel 2019 (Microsoft Corp.). Qualitative and quantitative analysis of the data was undertaken employing the Statistical Package for the Social Sciences Version 25 (IBM Corp.). Frequencies and percentages were used to present qualitative data, with analysis conducted through the χ^2^ test and Fisher's exact tests. For quantitative variables, the one-way ANOVA and Tukey's post-hoc analysis, when required, were utilized and the outcomes were expressed as means and standard deviations. P<0.05 was considered to indicate a statistically significant difference.

## Results

### Patient demographics and surgery indication

A total of 419 patients underwent LC from January 2021 to January 2023, of which females were the predominant gender (78.5%). The mean and median ages of the cases were 46.3±15.8 years and 45 years, with a range of 2-90 years, respectively. Their mean body mass index (BMI) was 29.5±5.1 kg/m^2^. The most common indication for surgery was biliary colic (BC) (69.5%), followed by acute cholecystitis (AC) (23.9%) (Table I).

### Main findings

Subtotal cholecystectomy was performed in two cases (0.48%). Identification of RS was achieved in 320 (76.4%) cases, comprising 60 (18.8%) males and 260 (81.2%) females. A history of previous surgery or previous admission and the BMI did not significantly affect the identification of RS (P>0.05). However, female gender, surgery indication for AC and BC, and adhesion significantly affected the identification of RS (P<0.05; Table II). The CVS was achieved in 383 (91.4%) cases and the RS was identified in 320 cases (76.4%), while both RS identification and CVS achievement were present in 322 patients (76.8%). The duration of the operation was significantly shorter in cases where at least the CVS (45.6±17.9 min) or RS identification (45.6±18.6 min) was achieved compared to those where achievement of the CVS (63.7±27.7 min) or RS identification (50.7±21.7 min) was unsuccessful, respectively (P<0.05). Surgeries for patients with both CVS achievement and RS identification combined were significantly shorter in duration (44.3±17.6 min) compared to those in which the combination of these factors was not observed (53.3±22.6 min; P<0.001). In addition, male gender, adhesion, GB size and wall thickness all significantly increased the operation time (P<0.05; Table III). On multiple-comparison analyses, the presence of AC significantly increased the time of operation in comparison to the presence of biliary colic and polyp (P<0.05); however, the other indications were not associated with a significantly different operation time among each other (P>0.05; Table IV).

### Complications and management

Among cases where neither CVS achievement nor RS identification were observed (97, 23%), eight patients (8.2%) had adhesions, 12 (12.4%) had a distended GB and 10 (10.3%) exhibited thick GB walls. In addition, in four patients (4.1%), GB perforation occurred, two patients (2.1%) experienced bleeding and one patient (1%) had stone spillage (Table V).

No conversion to open surgery was required in any of the cases. However, GB perforation occurred in 64 cases (15.3%), bleeding in 39 cases (9%) and stone spillage in 24 patients (5.7%). In 56 cases (13.4%), the placement of a drain was necessary. In all cases of bleeding, it was mild, requiring no blood transfusion, and it was controlled by cauterization. Five patients developed postoperative jaundice and their diagnosis revealed retained common bile duct stones (Table V). Among them, one patient underwent endoscopic retrograde cholangiopancreatography for stone extraction and stent insertion, while the remaining cases were managed conservatively (data not shown). Umbilical hernia developed in five female patients (Table V). Among these cases, four were managed using the open technique, while the VisiPort was employed in one case (14). None of the cases needed intensive care unit admission (data not shown).

## Discussion

Despite advances in resources, BDI remains a critical issue in LC. In a study conducted by Reinsoo *et al* (15) over 11 years, 241 BDIs were reported among 29,739 LCs (0.81%): 0.68% were minor injuries and 0.13% were major injuries. According to a systematic review and meta-analysis in China, the incidence rate of BDI was 1.12% (16). In a large study in India (17), it was 0.05%, and in a local study (Sulaimani, Kurdistan region, Iraq) (18), it was 0.4%. However, a study by Buddingh *et al* (4) from the Netherlands showed a significantly lower rate of major BDI after the implementation of routine IOC (1.9 vs. 0%).

In difficult cholecystectomy (DC), conversion to open procedure, duration of surgery, postoperative complications and reinterventions are taken into consideration (19). The difficulty of LC can arise from obscure anatomy, dense adhesions and unclear anatomy at CT, primarily attributed to the impact of the degree of inflammation and fibrosis on the dissection planes, or adhesions from previous surgery. These factors increase the risk of BDI. An experienced surgeon is required to operate on these patients, and if difficulties arise, conversion to an open procedure should be opted for. Parameters predicting difficult operations include male gender, upper abdominal tenderness at the time of surgery, a history of previous upper abdominal surgery, age of >60 years and a diagnosis of AC (20). However, DC can still be unpredictable due to the variability of operative findings (19). For this reason, several intra-operative difficulty scores for use in LC have been published. Nassar *et al* (21) considered the GB wall, adhesions and cystic pedicles, as these factors make grasping of the GB and dissection difficult. Another study included factors such as grasping the GB, adhesiolysis, CT dissection, anatomical anomalies and difficulty in GB extraction (22). Sugrue *et al* (23) took into account GB wall adhesions, BMI and the degree of infection.

Furthermore, the widespread utilization of the CVS serves as a valuable approach to prevent BDI (24,25). In the present study, the CVS was successfully achieved in 91.4% of the patients. The duration of operations for patients in whom the CVS was achieved was shorter (45.6±17.9 min) compared to those where the CVS was not achieved (63.7±27.7 min; P<0.001), as the target points made the procedure systematic and well-guided. Furthermore, an operation duration exceeding the average time may indicate the complexity of LC (26). Among the cases, 24.58% had AC, 35.5% had a thick-walled GB and 41% had a distended GB. This suggests that the CVS can be achieved more easily when there are fewer adhesions and less inflammation present. In a systematic review, the CVS was successfully achieved in 92.5%, and CVS-related BDI was reported at a rate of 0.09% (25). However, in a study by Nassar *et al* (27), the CVS was identified in 83% of consecutive LC cases. Within that study, AC was detected in 11.85% of patients, while BC was present in 60% of the patients. Furthermore, the RS landmark can serve as a consistent extra-biliary reference point for identifying the appropriate starting site for dissection during LC, aiding in the prevention of BDI. In a study conducted by Cheruiyot *et al* (6), the overall prevalence of RS was found to be 83%. Of note, there were no significant differences in the prevalence of RS between cadaveric studies (82%) and laparoscopic studies (83%). In the present study, the presence of RS was observed in 76.4% of cases. It has been reported that the presence of RS aids in the swift and straightforward identification of anatomical structures during LC, potentially leading to a reduction in surgical duration. A study from Nepal showed that the mean surgery duration for patients with identified RS was 29.16±8.7 min, whereas it was 42.9±23.6 min for those with no identified RS (28). Accordingly, in the present study, the operation time was significantly decreased in cases with identified RS (45.6±18.6 min) in comparison to those without RS identification (50.7±21.7 min).

Achievement of both the CVS and identification of RS was present in 322 cases (76.84%) and significantly reduced the operation time. In 36 patients, the CVS could not be achieved, and identification of the RS can be used as a protective finding in cases where the CVS cannot be achieved. Among the 419 patients, neither the CVS nor RS could be found in 97 cases (23%). Among these cases, eight patients (47%) had adhesions, 12 (70%) had a distended GB and 10 (58.8%) exhibited thick GB walls. In addition, GB perforation occurred in four patients, bleeding in two patients and stone spillage in one patient. The mean operation time for patients with distended GB was 53.3 min, as more time was required for dissection. However, through meticulous dissection and careful attention, the surgeries concluded without any postoperative complications. Subtotal cholecystectomy was performed in two cases, as it serves as a one-stage bail-out procedure for challenging cholecystectomies (24).

While conversion from LC to open surgery is not typically considered a failure, it is associated with significant morbidity. Therefore, it should be pursued only as a last resort when alternative strategies have proven unsuccessful (27). However, patient and surgeon-related factors and equipment failure can contribute to conversion. The primary reason for conversion is often attributed to challenges in visualizing the CVS and difficulties in dissection caused by unclear anatomy. In addition, intraoperative complications such as bleeding, BDI and intestinal perforation can lead to the need for conversion (29). Although in a local study (Sulaimani, Kurdistan region, Iraq), the CR was 4.5% (30), in the present study, no conversion to open surgery was required. However, there were instances of bleeding in 39 cases (9%), GB perforation in 64 cases (15.3%) and stone spillage in 24 patients (5.7%), where total cholecystectomy was completed. Furthermore, a drain was necessary in 56 cases (13.4%). In a study by Duca *et al* (3), GB perforation was 15.9%, while bleeding was 2.3%. In another study, the bleeding was 5.1% and they used drain in 26.5% of the cases (17), and others reported GB perforation in 4.9% of cases, bleeding in 0.4% and stone spillage in 2.5% (19). In a comprehensive study conducted by Nassar *et al* (27), the CR was reported at 0.49%. The study included challenging cases and involved bile duct exploration. In another study, it was reported to range from 1 to 23.3%, underscoring the significance of training and sub-specialization (31). Emphasizing a high case volume and employing strategies such as subtotal cholecystectomy can help reduce CR. In the present study, IOC was not employed, as a meta-analysis concluded that there is no statistically significant difference in BDI rates between routine and selective IOC groups. Furthermore, IOC is not always available. Therefore, exercising patience with meticulous dissection, enhancing vision and seeking the assistance of colleagues may serve as alternatives to IOC or other modalities for identifying anatomy or anomalies, aiming to prevent BDI (32). The prominent limitations of the present study are the small sample size, short follow-up period, not grading postoperative outcomes according to Clavien Dindo's classification and lack of data for comparative analysis of intraoperative and postoperative complications based on CVS status. Future studies that focus on the comparative analysis of intraoperative and postoperative complications of LC regarding the achievement or non-achievement of the CVS can provide a better understanding of the advantages associated with achieving CVS in LC.

In conclusion, achieving the CVS and identifying RS can serve as practical landmarks for ensuring the safety of LC and reducing the CR. Furthermore, employing meticulous dissection techniques and allocating sufficient time for dissection can be regarded as effective strategies for preventing BDI and minimizing the CR.

## Figures and Tables

**Table I:** Baseline characteristics of the participants (n=224).

Characteristic	Value
Age, years	46.3±15.8
Sex	
Male	90 (21.5)
Female	329 (78.5)
History of previous surgery	24 (5.7)
History of previous admission	37 (8.8)
Body mass index, kg/m^2^	29.5 (5.1)
Comorbidity	
Hypertension	40 (9.5)
Diabetes	12 (2.9)
Hypothyroidism	11 (2.6)
Others	21 (5.0)
Presence of Rouviere's sulcus	320 (76.4)
Indication of surgery	
Biliary colic	291 (69.5)
Acute cholecystitis	100 (23.9)
Pancreatitis	12 (2.9)
Polyp	12 (2.9)

Values are expressed as the mean ± standard deviation or n (%).

**Table II:** Association of sex, history of surgery and admission, BMI, surgery indication and adhesion with identification of Rouviere's sulcus.

	Rouviere's sulcus		
Characteristic	Identified	Not identified	P-value
Sex			0.017
Male	60 (18.8)	30 (30.3)	
Female	260 (81.2)	69 (69.7)	
History of surgery	15 (4.7)	9 (9.1)	0.085
History of admission	25 (7.8)	12 (12.1)	0.223
BMI	29.6 (5.0)	29.3 (5.3)	0.645
Surgery indication			
Biliary colic	232 (72.5)	59 (59.6)	0.018
Cholecystitis	68 (21.3)	35 (35.4)	0.007
Pancreatitis	12 (3.7)	1 (1.0)	0.309
Polyp	8 (2.5)	4 (4.0)	0.489
Adhesion	100 (68.5)	46 (31.5)	0.003

Values are expressed as n (%). BMI, body mass index.

**Table III:** Association of sex, identification of CVS and RS, previous surgery, indication for surgery, history of ERCP and GB characteristics with operation time.

Characteristic	Cases, n	Surgery duration, min	P-value
Sex			<0.001
Male	90	55.6±24.6	
Female	329	44.2±17.0	
CVS achievement			<0.001
Yes	383	45.6±17.9	
No	36	63.7±27.7	
Presence of RS			0.027
Yes	320	45.6±18.6	
No	99	50.7±21.7	
Presence of RS + CVS			<0.001
Yes	322	44.3±17.6	
No	97	53.3±22.6	
History of previous upper abdominal surgery			0.288
Yes	21	51.2±24.2	
No	398	46.5±19.2	
Indication for surgery			<0.001
Biliary colic	291	43.1±15.7	
Cholecystitis	103	58.3±24.6	
Pancreatitis	13	46.2±24.9	
Polyp	12	41.2±13.7	
History of ERCP			0.235
Yes	22	51.6±25.2	
No	397	46.5±19.1	
Adhesion			<0.001
Yes	140	56.4±23.3	
No	279	41.7±14.8	
GB size			<0.001
Normal	248	40.2±13.4	
Distended	171	53.3±21.5	
GB wall thickness			<0.001
Normal	270	40.8±13.7	
Thick	149	57.0±23.3	

Values are expressed as the mean ± standard deviation. CVS, critical view of safety; RS, Rouviere's sulcus; ERCP, endoscopic retrograde cholangiopancreatography; GB, gallbladder.

**Table IV:** Multiple comparisons of surgery indications regarding operation time (minutes).

Indication	Compared indication	Mean difference (indication- compared indication) ± standard error	95% Confidence interval	P-value
Biliary colic	Cholecystitis	-15.21±2.19	-(20.88-9.54)	<0.001
	Pancreatitis	-3.06±5.45	(-17.13-11.01)	0.944
	Polyp	1.94±5.45	(-12.13-16.01)	0.985
Cholecystitis	Biliary colic	15.21±2.19	(9.54-20.88)	<0.001
	Pancreatitis	12.15±5.66	(-2.47-26.77)	0.141
	Polyp	17.15±5.66	(2.52-31.77)	0.014
Pancreatitis	Biliary colic	3.06±5.45	(-11.01-17.13)	0.944
	Cholecystitis	-12.15±5.66	(-26.77-2.47)	0.141
	Polyp	5.0±7.55	(-14.49-24.49)	0.911
Polyp	Biliary colic	-1.94±5.45	(-16.01-12.13)	0.985
	Cholecystitis	-17.15±5.66	(-31.77-2.52)	0.014
	Pancreatitis	-5.0±7.55	(-24.49-14.49)	0.911

**Table V:** Intraoperative and post-operative complications.

A, Overall complications due to the procedure	
Item	n (%)
GB perforation	64 (15.3)
Liquid or air collection needs a drain	56 (13.4)
Bleeding	39 (9.0)
Stone spillage	24 (5.7)
B, Challenges in cases with no CVS and RS	
Distended GB	12 (12.4)
Thick GB walls	10 (10.3)
Adhesions	8 (8.2)
C, Complications in cases with no CVS and RS	
GB perforation	4 (4.1)
Bleeding	2 (2.1)
Stone spillage	1(1)
D, Postoperative diagnosis	
Jaundice caused by CBD stones	5 (1.2)
Umbilical hernia	5 (1.2)
GB cancer	1 (0.2)

CVS, critical view of safety; GB, gallbladder; CBD, common bile duct; RS, Rouviere's sulcus.

## Data Availability

The data generated in the present study may be requested from the corresponding author.
